# Hemoadsorption in a Case of Severe Septic Shock and Necrotizing Fasciitis Caused by Nontraumatic Renal Rupture due to Pyelonephritis with Obstructive Uropathy

**DOI:** 10.1155/2018/5248901

**Published:** 2018-04-29

**Authors:** Lampros Kousoulas, Uwe Wittel, Stefan Fichtner-Feigl, Stefan Utzolino

**Affiliations:** Department of General and Visceral Surgery, Medical Center, University of Freiburg Faculty of Medicine, Freiburg, Germany

## Abstract

**Background:**

Nontraumatic renal rupture due to pyelonephritis with obstructive uropathy is an uncommon but life-threatening situation.

**Case Presentation:**

A 25-year-old female presented to the emergency department with acute worsening of abdominal pain that began four weeks earlier. She was found to have peritonitis, leukocytosis, severe lactic acidosis, and a pronounced anemia and imaging was consistent with nontraumatic renal rupture with retroperitoneal abscess, perforation of the colon, and severe necrotizing fasciitis of the right lower limb. She underwent a right nephrectomy, a right hemicolectomy, surgical debridement of the retroperitoneum, and an upper thigh amputation. Due to severe septic shock and rhabdomyolysis with acute renal failure we performed a combined treatment of hemoadsorption using a Cytosorb hemoadsorber and continuous venovenous hemodialysis (CVVHD). Subsequently the patient recovered and was discharged home with no signs of infections and with normal renal function.

**Conclusion:**

We present a case of pyelonephritis with nontraumatic renal rupture leading to necrotizing fasciitis with osteomyelitis of the lower limb. The early treatment of the patient with a Cytosorb hemoadsorber led to a rapid hemodynamic and metabolic stabilization and preservation of the renal function, suggesting that hemoadsorption might be a rescue therapy in patients with severe septic shock and traumatic rhabdomyolysis.

## 1. Clinical History

We report the case of a 25-year-old female patient with no history of surgery who presented to the emergency department with acute worsening of abdominal pain that began four weeks prior. Her pain was located in the right lower quadrant of her abdomen, was exacerbated by movement, and was radiating to her right lower limb. The patient reported having suffered from recurrent urinary tract infections that were treated by her physician. There was no referral to urology or microbiological evaluation.

## 2. Physical Examination and Diagnosis

Physical examination upon arrival revealed a Glasgow Coma Scale of 15, blood pressure of 100/50 mmHg, heart rate of 135 beats per minute with regular rhythm, normal blood oxygen saturation, and a body temperature of 38°C. Her abdomen was slightly distended and deep palpation of the right lower abdominal quadrant caused pain. Moreover, the patient could not move her right leg due to pain. The rest of her physical examination was unremarkable. Blood analysis revealed leukocytosis (19,000/*μ*l) and a pronounced anemia with a hemoglobin of 3.5 g/dl. Blood chemistry revealed 332 mg/l C-reactive protein (CRP) and 15.95 ng/ml procalcitonin (PCT), elevated lactate of 4.3 mmol/L, and elevated lipase (1524 U/L). Urine analysis showed a pH 5.0 and many white cells per field under high-power magnification.

**Figure 1:**
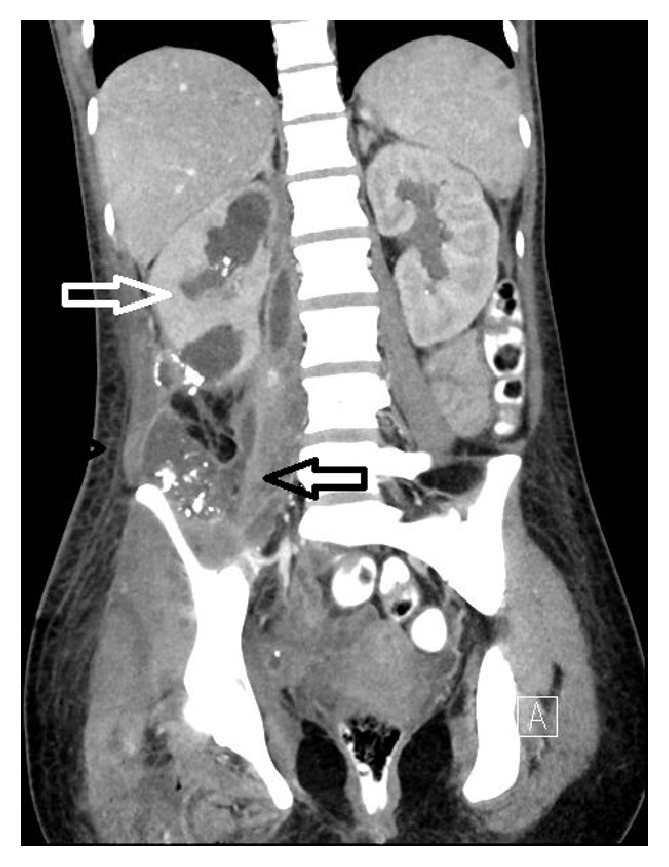
Computed tomography shows a completely disrupted right kidney with multiple stones (white arrow) and a large retroperitoneal abscess (black arrow).

**Figure 2:**
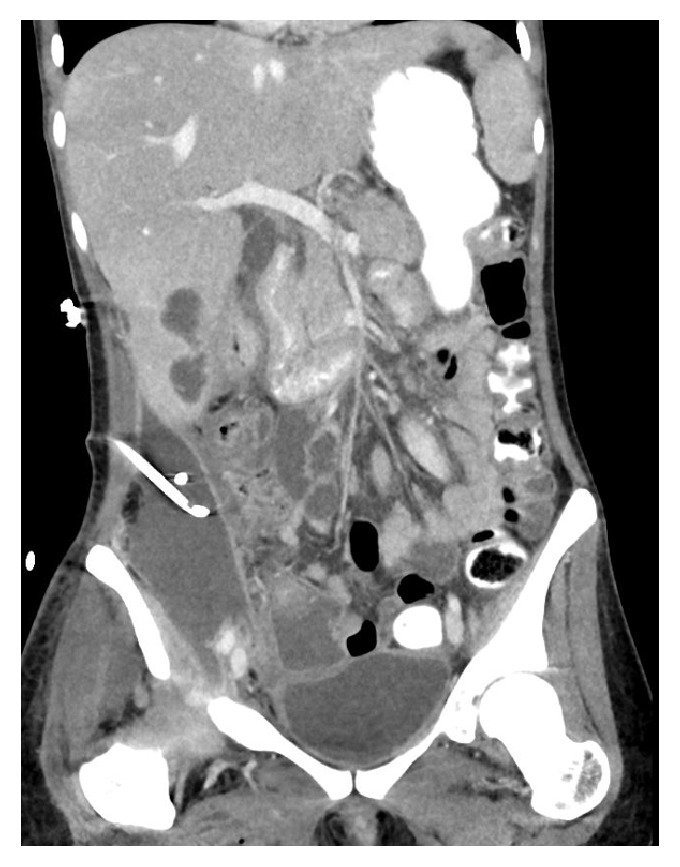
Computed tomography shows the retroperitoneal abscess after the insertion of the transcutaneous drainage.

Abdominal sonography revealed presence of an abscess extending down to the right lower abdomen and the patient underwent transcutaneous drainage of the abscess. Due to an apparently intestinal secretion, an abdominal computed tomography (CT) was performed exposing a completely disrupted right kidney with multiple stones and a large retroperitoneal abscess extending down to her right lower limb, as well as a cecal perforation. We diagnosed this case as a nontraumatic renal rupture due to pyelonephritis with obstructive uropathy with stones leading to a retroperitoneal abscess with inflammatory perforation of the colon and severe necrotizing soft tissue infection of the right lower limb (Figures 1 and 2). The patient was admitted to the surgical intensive care unit (ICU) and had by admission a calculated SAPS II score of 29. After initial resuscitation and intravenous administration of piperacillin/tazobactam 4/0.5 g, she was scheduled for emergency surgical intervention.

## 3. Intervention

An exploratory laparotomy was performed revealing diffuse retroperitoneal purulence, as well as an abscess rupture of the right kidney and a perforation of the colon at the base of the caecum. The patient underwent a partial right nephrectomy and a right hemicolectomy as well as surgical drainage of the retroperitoneal abscess. We also performed one incision on the medial right thigh and one incision of the calf resulting in the drainage of pus.

The patient was postoperatively transferred to the ICU and had postoperatively a calculated SAPS II score of 32. She remained hemodynamically unstable and laboratory testing revealed severe lactic acidosis. She continued to require vasopressor support and end organ damage was evident on laboratory findings. Due to the presence of severe septic shock, the initial antibiotic regimen of intravenous piperacillin/tazobactam was changed to imipenem/cilastatin and linezolid as well as antifungal therapy with micafungin. The abscess cultures showed presence of* E. coli*,* Klebsiella oxytoca*,* Clostridium ramosum*,* Bacteroides vulgatus*,* Candida glabrata*, and* Candida dubliniensis*, whereas the blood cultures of the patient showed presence of* Candida glabrata*. The urine cultures of the patient showed presence of* Klebsiella oxytoca*.

**Figure 3:**
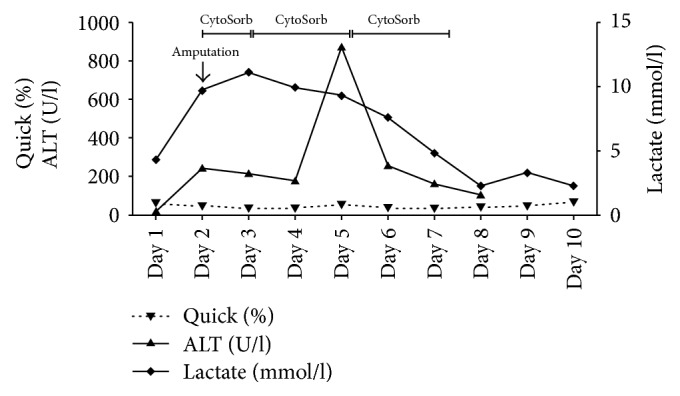
Plasma concentrations of Quick, ALT, and Lactate during the course of the three Cytosorb sessions. Day 1 represents start of treatment after postoperative transfer to the ICU (ALT: alanine transaminase).

**Figure 4:**
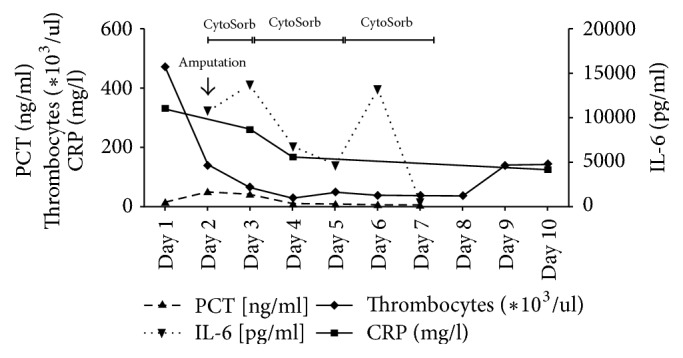
Plasma concentrations of PCT, Thrombocytes, CRP, and IL-6 during the course of the three Cytosorb sessions. Day 1 represents start of treatment after postoperative transfer to the ICU.

**Figure 5:**
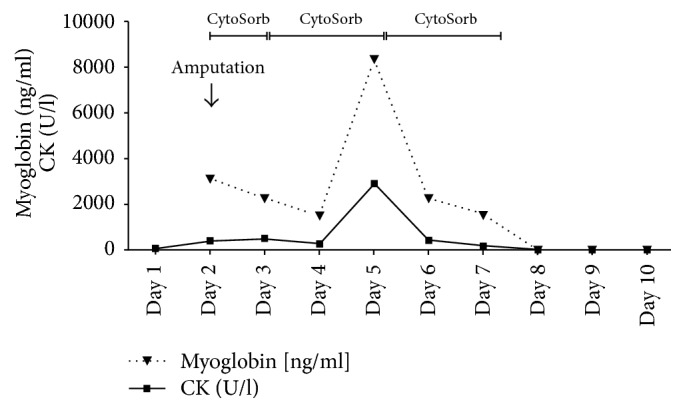
Plasma concentrations of Myoglobin and CK during the course of the three Cytosorb sessions. Day 1 represents start of treatment after postoperative transfer to the ICU (CK: Creatin-Kinase).

**Figure 6:**
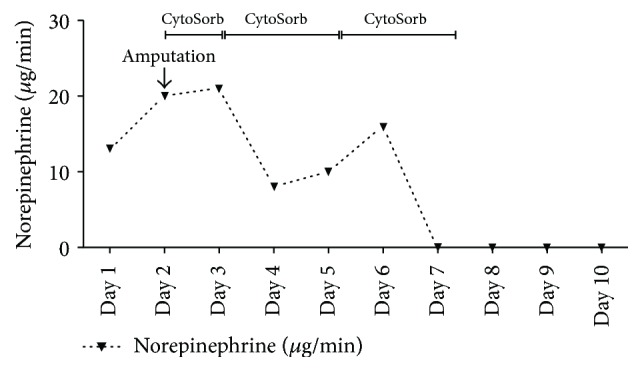
Shown is the need of vasopressors (Norepinephrine) during the course of the three Cytosorb sessions. Day 1 represents start of treatment after postoperative transfer to the ICU.

Postoperatively, the patient developed a traumatic rhabdomyolysis with acute renal failure, fulminant hepatic failure, and severe lactic acidosis (Figure 3) accompanied by extensively elevated interleukin 6 (IL-6) and myoglobin levels (IL-6: 10796 pg/ml, myoglobin: 3126 ng/ml). We performed a combined treatment of hemoadsorption using a Cytosorb hemoadsorber (CytoSorb; Cytosorbents GmbH, Berlin, Germany) and continuous venovenous hemodialysis (CVVHD) (Multifiltrate, Fresenius Medical Care) while the patient had to be mechanically ventilated suffering from acute respiratory distress syndrome. Septic shock persisted without clinical or laboratory improvement and IL-6 levels rose (IL-6: 13712 pg/ml) so that the patient was again taken to the operating room, where residual right nephrectomy was performed. The surgical exploration of the right thigh showed a severe necrotizing fasciitis with osteomyelitis so that an upper thigh amputation had to be carried out. Because of the anticipation of the need for at least one more surgical revision, a negative pressure wound therapy system was applied and the patient was transferred to the ICU. Responding to a recurring clinical deterioration with new elevation of IL-6 levels, we performed a surgical revision of the right thigh with local resection due to progression of necrotizing fasciitis. Postoperatively, the IL-6 and myoglobin levels increased (IL-6: 13279 pg/ml, myoglobin: 8365 ng/ml) and so two more cycles of Cytosorb therapy, each for 48 hours, were performed in order to attenuate the inflammatory response and reduce the myoglobin levels (Figures 4 and 5). Subsequently the patient recovered, vasopressor therapy could be stopped (Figure 6), the IL-6 and myoglobin levels returned to normal, and the kidney and liver function were stabilized. Two days later, the patient was successfully extubated accompanied by further improvement in renal function and discontinuation of hemodialysis. Forced diuresis was performed due to pronounced edema and, after successful negative balancing, we removed the negative pressure wound therapy system and closed the abdominal wall of the patient. Based on the antibiogram, the initial antibiotic therapy was changed to ampicillin/sulbactam, whereas the antimycotic therapy with micafungin was continued. Two weeks after the initial operation, the patient's blood tests revealed elevated leukocytes and CRP, so that an abdominal CT was performed. The CT scan showed impaired peripheral perfusion of the liver with ischemic necrosis of liver segments V–VIII and infected parahepatic hematoma. She underwent transcutaneous drainage of the hematoma and was started on long-term oral therapy with posaconazole as the cultures showed presence of* Candida glabrata* and* Candida dubliniensis*.

## 4. Follow-Up

Finally, after 35 days of hospital stay the patient was discharged home with no signs of infections and with normal renal function, with plans for outpatient physical therapy and being on medication with pregabalin due to mild phantom pain.

## 5. Discussion

Renal rupture most often occurs as a result of traumatic injury [1]. Nontraumatic renal rupture is a rare condition, with only case reports and small case series being reported in the literature. The majority of patients with nontraumatic renal rupture have a renal tumor [2, 3] but also some other conditions such as obstructive uropathy with stones, pyelonephritis with abscess, aneurysm, infarct, and autoimmune disorders can be the cause [4, 5]. Patients present with flank pain, hematuria, and anemia. A conservative approach with selective angiography and arterial embolization in stable patients may be successful, but a nephrectomy via a midline transabdominal approach is the treatment of choice [6]. In our case there was a history of recurrent urinary tract infections leading to a complete disruption of the right kidney with retroperitoneal abscess, severe necrotizing fasciitis, and osteomyelitis of the right lower limb, which are extremely rare complications of pyelonephritis.

Necrotizing fasciitis is a deep-seated infection of subcutaneous tissue that is characterized by progressive necrosis of the fascia and fat [7]. Necrotizing fasciitis may result from any injury to the skin or from hematogenous spread and treatment by a combination of surgical debridement, appropriate antibiotics, and optimal oxygenation of the tissue is mandatory and the need for amputation is rare [8]. In this patient the protracted course led to later histologically confirmed osteomyelitis, which also is a rare finding in necrotizing fasciitis. In our patient the septic shock resolved only after the upper thigh amputation was performed.

Because of severe traumatic rhabdomyolysis with renal failure we performed three cycles of hemoadsorption using a Cytosorb hemoadsorber with the intention to remove myoglobin and maintain the renal function. Treatment was associated with a rapid hemodynamic stabilization and a decrease in IL-6 as well as myoglobin levels, which is one of the main effects to be expected from the Cytosorb hemoadsorber [9, 10]. It is important to mention that the use of a Cytosorb hemoadsorber attenuated the inflammatory response, reduced the IL-6 and myoglobin levels, and led to a significant hemodynamic stabilization of the patient, only after adequate surgical treatment of the septic focus. The IL-6 levels of the patient rose twice under the Cytosorb therapy and only after the surgical revision of the right thigh and the performance of the amputation did the septic shock resolve.

In conclusion, we present a rare case of severe pyelonephritis with nontraumatic renal rupture leading to necrotizing fasciitis with osteomyelitis of the lower limb. The early treatment of the patient with a combination of CVVHD and Cytosorb led to a rapid hemodynamic and metabolic stabilization and preservation of the renal function, suggesting that hemoadsorption might be a rescue therapy option in patients with severe septic shock and traumatic rhabdomyolysis.
